# Patient experience with outpatient encounters at public hospitals in Shanghai: Examining different aspects of physician services and implications of overcrowding

**DOI:** 10.1371/journal.pone.0171684

**Published:** 2017-02-16

**Authors:** Yuhua Bao, Guanrong Fan, Dongdong Zou, Tong Wang, Di Xue

**Affiliations:** 1 Departments of Healthcare Policy & Research and Psychiatry, Weill Cornell Medical College, New York, New York, United States of America; 2 Shanghai Medical Ethos Association, Shanghai, China; 3 Shanghai Health and Family Planning Commission, Shanghai, China; 4 Department of Hospital Management, School of Public Health and Key Laboratory of Health Technology Assessment, Fudan University, Shanghai, China; University of Rochester, UNITED STATES

## Abstract

**Background:**

Over 90% of outpatient care in China was delivered at public hospitals, making outpatient experience in this setting an important aspect of quality of care.

**Objective:**

To assess outpatient experience with different aspects of physician services at China’s public hospitals and its association with overcrowding of the hospital outpatient departments.

**Research design:**

Retrospective analysis of a large survey of outpatient experience in Shanghai, China. We tested the hypotheses that patient experience was poorer with physician-patient communication, education, and shared decision-making and where and when there was greater overcrowding of the hospital outpatient departments. Ordered logistic models were estimated separately for general and specialty hospitals.

**Subjects:**

7,147 outpatients at 40 public hospitals in Shanghai, China, in 2014.

**Measures:**

Patient experience with physician services were self-reported based on 12 questions as part of a validated instrument. Indicators of overcrowding included time of visit (morning vs. afternoon, Monday vs. rest of the week) and hospital outpatient volume in the first half of 2014.

**Results:**

Overall, patients reported very favorable experience with physician services. Two out of the 12 questions pertaining to both communication and shared decision-making consistently received lower ratings. Hospitals whose outpatient volumes were in the top two quartiles received lower patient ratings, but the relationship achieved statistical significance among specialty hospitals only.

**Conclusions:**

Inadequate physician-patient communication and shared decision-making and hospital overcrowding compromise outpatient experience with physician services at Chinese public hospitals. Effective diversion of patients with chronic and less complex conditions to community health centers will be critical to alleviate the extreme workloads at hospitals with high patient volumes and, in turn, improve patient experience.

## Introduction

Patient experience during clinical encounters with their providers is an important aspect of quality of care [1]. Many argue that the intrinsic values of patient experience–the expectation of humane, empathic care that actively involves patients in clinical decision-making–requires no further justifications [2]. On the other hand, there is cumulating evidence that better patient experience was associated with improved adherence to medications and treatments, improved clinical and other health outcomes, and greater safety and reduced adverse events [3, 4]. Measures of patient experience are thus increasingly adopted in public reporting and pay-for-performance programs in major health care systems. Prominent examples include various programs of the U.S. Centers for Medicare and Medicaid Services to incentivize quality of care by hospitals, physicians, and health care systems [5] and U.K.’s Commissioning for Quality and Innovation payment framework [6].

Patient experience with outpatient care is becoming an increasingly salient aspect of quality of care at public hospitals in China. Unlike in most developed countries where the bulk of outpatient care takes place at private physician offices, in China, 90% of all outpatient visits in 2012 were delivered at outpatient departments at public hospitals [7]. Over-crowding, long wait times, and brief encounters with the physicians characterize outpatient care at large public hospitals in China, known as the Tier 2 and Tier 3 hospitals [8]. Tier 2 hospitals are typically general hospitals affiliated with a medium size city, a county, or a district of a large city and have a bed size between 100 and 500 [9]. Tier 3 hospitals typically serve as regional medical hubs, are general or specialty hospitals, and have a bed capacity exceeding 500 [9]. Despite recent efforts to build up the capacity of primary and preventive care at community health centers (Tier 1 hospitals) and to divert patients with less complex needs to these community settings, the outpatient capacities at Tiers 2 and 3 hospitals remain strained. Average daily volumes of outpatient visits in 2014 to Tier 1, Tier 2, and Tier 3 general hospitals in Shanghai–one of the most populous and economically advanced cities in China–were 2,896, 3,953, and 9,097, respectively (data from the Shanghai Health and Family Planning Commission). Physicians at Tier 2 and 3 hospitals typically see 40–50 outpatients during a work shift of 4 hours.

The importance of attending to patients’ experience with clinical encounters is further highlighted by the rapid increase in medical disputes in China that, in a few extreme cases, have resulted in violence towards medical providers [10, 11]. Data from the Chinese Hospital Management Association show that medical disputes in China have increased at an alarming annual rate of 23% since 2002 [12, 13]. These disputes center around areas of medical care such as diagnosis, surgery, and medication use [10] that are susceptible to errors or disputes resulting from poor physician-patient communication and inadequate patient engagement in clinical decision-making. Strained resources, and, in particular, the pressure to accommodate large volumes of outpatient visits at Tiers 2 and 3 public hospitals, may have contributed directly to poor patient experience with their outpatient visits [12, 14].

In this study, we examined patient experience during outpatient encounters with physicians at public hospitals in Shanghai, China. We used unique data from a large survey covering 40 public hospitals in 2014. We hypothesize that patient experience is poorer with physician-patient communication and shared decision-making, compared to other aspects of physician services such as physician respect for patients and technical aspect of care. We also hypothesize that patient experience is poorer when visits take place at a time or place characterized by overcrowding of the hospital outpatient department.

## Methods

This study was approved by the Institutional Review Board of Fudan University School of Public Health.

### Data and sample

We used data from an outpatient experience survey conducted at forty public hospitals (eight Tier 1, four Tier 2, and twenty-eight Tier 3) in the central city of Shanghai in July and August of 2014. The survey included all Tier 3 public hospitals under the administration of the Shanghai Health and Family Planning Commission except one mental health center and one hospital specializing in infectious diseases. Of the twenty-eight Tier 3 hospitals included, fifteen were general hospitals and thirteen were medical specialty hospitals; all these hospitals were located in urban, densely populated, and well-developed districts in the city center. Because socio-demographics of residents vary substantially between the central and more peripheral areas of Shanghai, to ensure comparability of patients, the survey also included eight Tier 1 hospitals and four Tier 2 hospitals located in the same districts as the Tier 3 hospitals in the study. All Tiers 1 and 2 hospitals are general hospitals.

A workweek was selected for each hospital during which a target of 200 outpatients were recruited to participate in the anonymous patient experience survey. Four or five volunteers were assigned to each hospital to recruit patients, obtain oral consent for study participation, and assist survey participants with completing the iPad-based survey. Volunteers were mainly medical students from the major medical colleges in Shanghai and received training on study protocols.

### Measures

The survey assessed experience of the patient during the index outpatient visit to a public hospital along four dimensions: facilities and equipment, physician services, non-physician services, and ancillary processes and effectiveness. Survey questions were initially based on a literature review of validated and widely adopted patient experience instruments [15–17] and consultation with local experts in hospital management. A pilot test of the questionnaire was conducted in 2013 among 5,569 outpatients at three Tier 3 hospitals, five Tier 2 hospitals and eight community health centers (Tier 1); psychometric analysis using the pilot data indicated high construct validity based on standard tests of goodness of fit in structural equation models [18, 19] and high internal reliability for all questions included (overall Cronbach’s alpha = 0.97). We provided goodness of fit statistics and internal reliability measures for individual dimensions as well as the entire questionnaire for both the 2013 data (pilot) and the 2014 data (used in this study) in S1 Text.

Our focus in this study is on the physician service dimension of patient’s experience, assessed using a set of 12 questions covering several aspects of patient encounters with the physician including physician respect for the patient, technical aspect of care, physician-patient communication, and engagement of patient in shared decision-making (Tables 1 and 2). Patients were asked to rate the extent to which they agreed with each statement using a 6-point scale: strongly disagree, disagree, somewhat disagree, somewhat agree, agree, or strongly agree. Patient ratings were highly skewed to the left, with 80–90% of questions rated with “agree” or “strongly agree”. We thus further collapsed the 6-level responses into three ordered categories: “somewhat agree” or lower, “agree”, or “strongly agree” (Tables 1 and 2).

**Table 1:** Patient experience with outpatient encounters at public hospitals in Shanghai: General Hospitals.

Item	Aspect	Somewhat agree or less		Agree		Strongly agree		Missing	
		n	%	n	%	n	%	n	%
**Courteous**: Physician was courteous.	Respect	311	6.6	1,169	24.8	3,234	68.5	5	0.1
**Listen**: Physician carefully listened to the patient.	Communication	323	6.8	1,173	24.9	3,210	68.0	13	0.3
**Ask**: Physician actively asked questions to better understand the patient’s situation.	Communication	552	11.7	1,205	25.5	2,926	62.0	36	0.8
**Exam**: Physician carefully conducted physical examination.	Care	420	8.8	1,149	24.4	3,066	65.0	84	1.8
**Explain**: Physician adequately explained diagnoses.	Communication	417	8.9	1,215	25.8	3,027	64.1	60	1.3
**Alternatives**: Physician provided detailed information regarding the treatment plan including alternative treatments.	Shared decision-making	510	10.8	1,228	26.0	2,863	60.7	118	2.5
**Answer**: Physician gave satisfactory answers to the patient’s questions.	Communication	384	8.2	1,189	25.2	3,093	65.5	53	1.1
**Consider**: Physician considered the patient’s preferences and situations when making treatment decisions.	Shared decision-making	381	8.1	1,200	25.4	2,977	63.1	161	3.4
**Privacy**: Physician protected patient privacy when providing medical care.	Respect	242	5.0	1,020	21.6	3,124	66.2	333	7.1
**Tests**: Physician adequately explained the results of tests and exams to the patient.	Communication	324	6.9	1,209	25.6	3,058	64.8	128	2.7
**Medication**: Physician explained to patient how to take prescribed medications and made suggestions for a healthier lifestyle.	Communication	452	9.6	1,139	24.1	2,978	63.1	150	3.2
**Understand**: Physician helped the patient better understand his or her health problems.	Shared decision-making	376	8.0	1,205	25.5	3,085	65.4	53	1.1
**Total**	** **	4,692	8.3	14,101	24.9	36,641	64.7	1,194	2.1

**Table 2:** Patient experience with outpatient encounters at public hospitals in Shanghai: Specialty Hospitals.

Item	Aspect	Somewhat agree or less		Agree		Strongly agree		Missing	
		n	%	n	%	n	%	n	%
**Courteous**: Physician was courteous.	Respect	191	7.9	738	30.4	1,494	61.5	5	0.2
**Listen**: Physician carefully listened to the patient.	Communication	199	8.2	788	32.5	1,429	58.9	12	0.5
**Ask**: Physician actively asked questions to better understand the patient’s situation.	Communication	336	13.9	730	30.1	1,345	55.4	17	0.7
**Exam**: Physician carefully conducted physical examination.	Care	258	10.7	714	29.4	1,430	58.9	26	1.1
**Explain**: Physician adequately explained diagnoses.	Communication	270	11.2	730	30.1	1,401	57.7	27	1.1
**Alternatives**: Physician provided detailed information regarding the treatment plan including alternative treatments.	Shared decision-making	326	13.5	735	30.3	1,291	53.2	76	3.1
**Answer**: Physician gave satisfactory answers to the patient’s questions.	Communication	266	11.0	720	29.7	1,418	58.4	24	1.0
**Consider**: Physician considered the patient’s preferences and situations when making treatment decisions.	Shared decision-making	249	10.3	717	29.5	1,380	56.8	82	3.4
**Privacy**: Physician protected patient privacy when providing medical care.	Respect	166	6.9	680	28.0	1,483	61.1	99	4.1
**Tests**: Physician adequately explained the results of tests and exams to the patient.	Communication	217	8.9	750	30.9	1,344	55.4	117	4.8
**Medication**: Physician explained to patient how to take prescribed medications and made suggestions for a healthier lifestyle.	Communication	263	10.9	717	29.5	1,306	53.8	142	5.9
**Understand**: Physician helped the patient better understand his or her health problems.	Shared decision-making	248	10.2	762	31.4	1,369	56.4	49	2.0
**Total**	** **	2,989	10.3	8,781	30.1	16,690	57.3	676	2.3

We constructed several indicators of hospital outpatient department over-crowding as proxies for time constraints faced by physicians when interacting with patients. The first indicator was a dichotomous indicator of whether the index visit occurred in the morning (vs. afternoon). Outpatient departments at public hospitals in China operate on a first-come-first-serve basis and are known to be more crowded in the morning than in the afternoon. The second indicator was day of the week (Monday vs. Tuesday-Friday) when the index visit took place. Weekend staffing at public hospitals in China is typically much lower than weekdays and only emergency care is available on Sundays. As a result, Mondays usually see a surge in volume of outpatients. (We collapsed Tuesdays-Fridays after an earlier analysis indicated that the coefficients did not differ significantly.) The third indicator was the volume of outpatient visits at the index hospital in the first half of 2014, categorized into quartiles among the twenty-seven general hospitals (Tiers 1–3) and the thirteen specialty hospitals (all Tier 3) in the study separately.

### Statistical analysis

Analysis was conducted first to describe patient ratings based on the original 6-point scale. To test hypotheses, we estimated ordered-logistic regressions of the categorical ratings (“somewhat agree” or lower, “agree”, or “strongly agree”) by pooling responses to all 12 questions, using patient-question as the unit of analysis. These analyses were conducted separately for general and specialty hospitals. Because patient experience questions were nested within outpatient visits and visits nested within hospitals, we used STATA’s survey commands to estimate robust standard errors by specifying the multi-level clustering [20].

We included as independent variables eleven dichotomous indicators of the questions (using the first question, physician courtesy, as the reference) and the three indicators of outpatient department overcrowding. All regression analyses also controlled for the following patient characteristics that may have affected patient expectations for or experience with physician services: patient gender, age, monthly household income (<5k, 5-10k, >10k RMB, or, <$750, $750–1,500, >$1,500 based on current exchange rate), education of the survey respondent (less than high school, high school, some college, college or above), whether a Shanghai resident (yes/no), self-reported health status (excellent/very good vs. good/fair/poor), and whether the patient was visiting the hospital for the first time or for a new condition (vs. for an existing condition). In addition, the analysis of general hospitals controlled for the medical specialty the patient sought care from (internal medicine/pediatrics/OB&GYN, medical specialty, or surgery) and the tier of the hospital (Tier 2, 3, vs. 1). The analysis of specialty hospitals controlled for the type of hospitals (pediatric, OB&GYN, cancer/lung, and others).

Outpatient volumes of all Tier 3 general hospitals were in the top two quartiles while almost all Tiers 1 and 2 hospitals were in the bottom two quartiles. The differences observed between different volume quartiles may reflect differences in quality of care or patient mix at hospitals of different tiers. We thus conducted a sensitivity analysis by restricting the general hospital analysis to Tier 3 hospitals.

## Results

Our analytical sample contained responses by 7,147 patients to the physician service domain of the patient experience survey, of which 4,719 were patients seeking outpatient care at general hospitals, and 2,428 were at specialty hospitals. Descriptive statistics of the samples are presented in Tables 3 and 4 for outpatients at general hospitals and specialty hospitals, respectively.

**Table 3:** Descriptive statistics of outpatients at public hospitals in Shanghai: General hospitals.

Characteristic (N = 4,719)	N (%)
Female	3,152 (66.79)
Age– 0–17	137 (2.90)
18–39	1,082 (22.93)
40–59	1,486 (31.49)
60-	2,014 (42.68)
Income–<5K RMB	1,883 (39.90)
5-10K RMB	2,017 (42.74)
>10K RMB	819 (17.36)
Education–High School	1,384 (30.05)
Some College	1,369 (29.73)
College or Above	1,852 (40.22)
Non-resident of Shanghai	525 (11.13)
Self-Reported Health–Excellent/Very Good	2,590 (54.88)
Good/Fair/Poor	2,129 (45.12)
New Patient or New Condition	1,230 (26.06)
Department–Internal Medicine	1,877 (39.78)
Medical Specialty	1,730 (36.66)
Surgery	751 (15.91)
Tier 1 Hospital	1,005 (21.30)
Tier 2 Hospital	687 (14.56)
Tier 3 Hospital	3,027 (64.14)
Morning (vs Afternoon) Visit	3,455 (73.21)
Monday (vs Tuesday-Friday) Visit	1,021 (21.64)
Hospital Outpatient Volume– 1^st^ Quartile	937 (19.86)
2^nd^ Quartile	1,133 (24.01)
3^rd^ Quartile	1,444 (30.60)
4^th^ Quartile	1,205 (25.54)

**Table 4:** Descriptive statistics of outpatients at public hospitals in Shanghai: Specialty hospitals

Characteristic (N = 2,428)	N (%)
Female	1,468 (60.46)
Age– 0–17	657 (27.06)
18–39	643 (26.48)
40–59	658 (27.10)
60-	470 (19.36)
Income–<5K RMB	675 (27.80)
5-10K RMB	1,101 (45.35)
>10K RMB	652 (26.85)
Education–High School	1,023 (43.57)
Some College	487 (20.74)
College or Above	838 (35.69)
Non-resident of Shanghai	631 (25.99)
Self-Reported Health–Excellent/Very Good	1,624 (66.89)
Good/Fair/Poor	804 (33.11)
New Patient or New Condition	860 (35.42)
Type of Specialty Hospital–Pediatric	591 (24.34)
OB&GYN	382 (15.73)
Lung/Cancer	569 (23.43)
Other Specialty	886 (36.49)
Morning (vs Afternoon) Visit	1,509 (62.15)
Monday (vs Tuesday-Friday) Visit	528 (21.75)
Hospital Outpatient Volume– 1^st^ Quartile	714 (29.41)
2^nd^ Quartile	607 (25.00)
3^rd^ Quartile	590 (24.30)
4^th^ Quartile	517 (21.29)

As shown in Table 1, among general hospital outpatients, over 60% of sampled patients indicated that they “strongly agreed”, and about 25% “agreed”, with each statement; about 15% of the entire sample provided a rating that was “somewhat agreed” or lower. However, there were noticeable differences between items. In particular, two items were associated with relatively higher percentages for “somewhat agree” or lower but relatively lower percentages in the top category, “strongly agree”, indicating poorer patient experience. These two items were “Physician actively asked questions…” (“ask”) and “Physician provided detailed information regarding the treatment plan including alternative treatments” (“alternatives”), reflecting the aspects of physician-patient communication and shared decision-making. For outpatients to specialty hospitals, while the overall distribution of responses were similar to that for general hospitals, two items in addition to “ask” and “alternatives”–“Physicians adequately explained the results of tests and exams to the patients” (“tests”) and “Physicians explained to patient how to take prescribed medications and made suggestions for a healthier lifestyle” (“medication”)–were also associated with lower ratings (Table 2).

**Fig 1:**
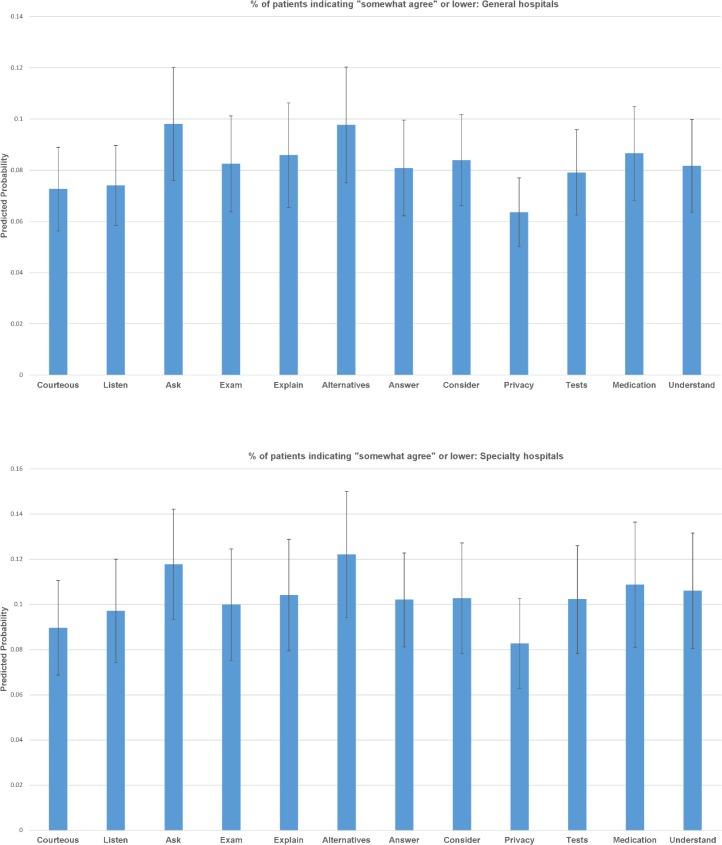
% of patients indicating “somewhat agree” or lower. (A) General hospitals. (B) Specialty hospitals.

**Fig 2:**
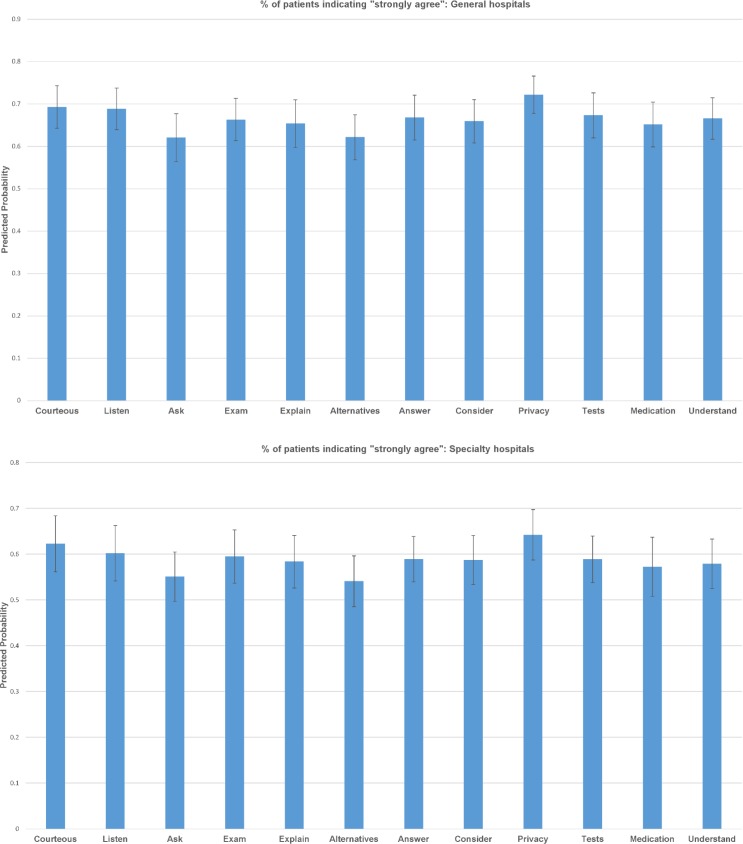
% of patients indicating “strongly agree.” (A) General hospitals. (B) Specialty hospitals.

Based on the ordered logistic regressions, we derived predicted probabilities (and 95% confidence intervals) of patient-rated experience for each of the 12 items. Largely consistent with the descriptive results, two items, “ask” and “alternatives”, received the least favorable ratings among all items in general hospitals. About 10% of all patients reported in the bottom four categories for these two items compared to 6–8% of all other items (Fig 1A). Consistently, 65% responses to these two items were in the top category compared to 70% or higher for other items (Fig 2A). None of these differences achieved statistical significance. A similar pattern held true for patient ratings at specialty hospitals (Figs 1B and 2B). For both general and specialty hospitals, the two items reflecting physician respect for patients, “Courteous” and “Privacy” consistently received higher ratings than other items.

**Fig 3:**
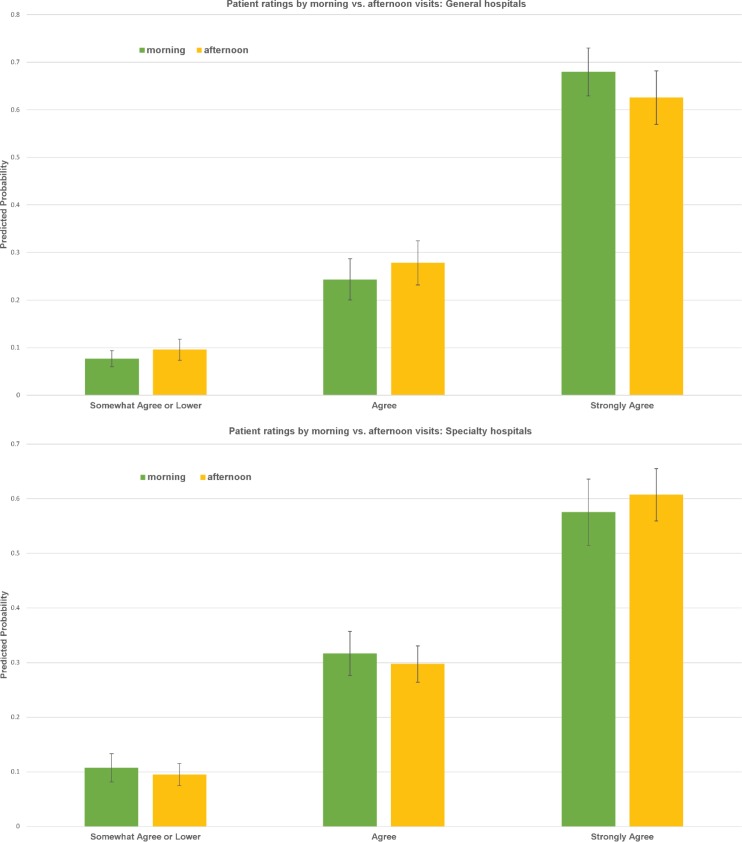
Patient ratings by morning vs. afternoon visits. (A) General hospitals. (B) Specialty hospitals.

**Fig 4:**
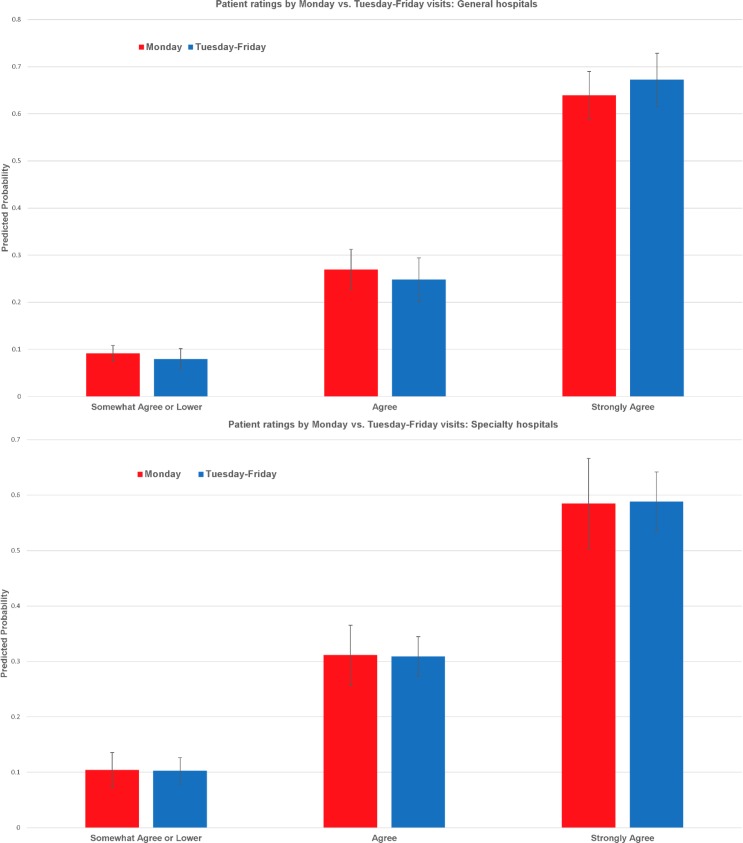
Patient ratings by Monday vs. Tuesday-Friday visits. (A) General hospitals. (B) Specialty hospitals.

**Fig 5:**
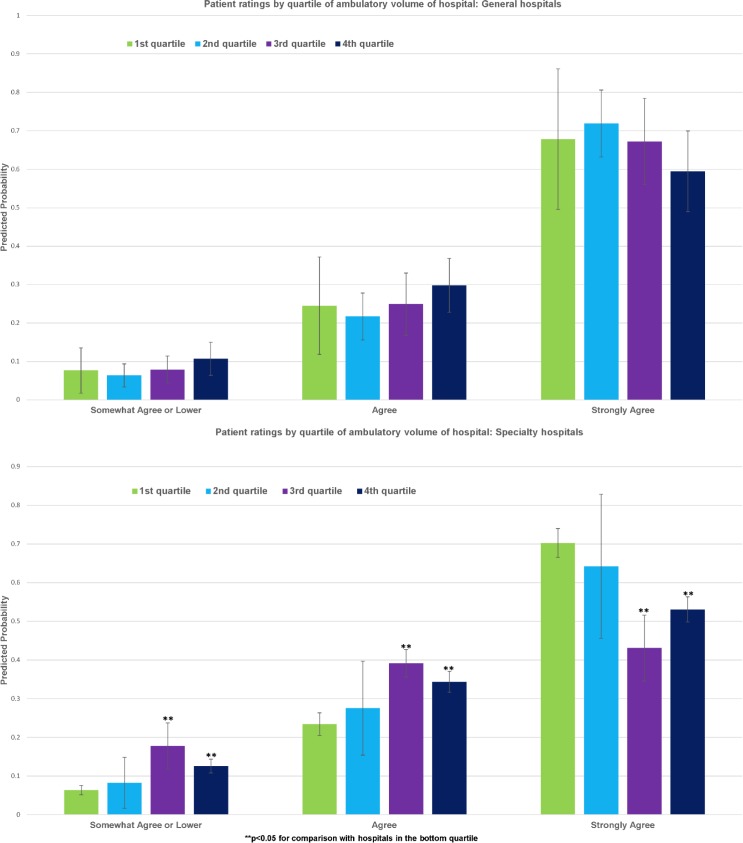
Patient ratings by quartile of ambulatory volume of hospital. (A) General hospitals. (B) Specialty hospitals. p<0.05 for comparison with 1^st^-quartile hospitals.

At both general and specialty hospitals, morning visits were associated with slightly lower ratings, compared to afternoon visits (Fig 3A and 3B), although none of the differences achieved statistical significance. Predicted ratings also did not reveal significant differences for Monday vs. Tuesday-Friday visits for general or specialty hospitals (Fig 4A and 4B). Results indicated that patient ratings at general hospitals became less favorable as hospital outpatient volume increased, especially among hospitals in top three quartiles in terms of outpatient volume (Fig 5A). At specialty hospitals, while differences were not statistically significant between hospitals in the 1st and 2nd quartiles in terms of outpatient volume, patients at hospitals in the 3^rd^ and 4^th^ quartiles were substantially more likely to have indicated “somewhat agree” or lower or “agree”, and substantially less likely to have indicated “strongly agree” (40–50% as opposed to 70%), compared to patients at hospitals in the first quartile (Fig 5B).

Sensitivity analysis by restricting the general hospital sample to Tier 3 hospitals generated similar results (S1–S3 Figs): point estimates suggested that each indicator of outpatient department overcrowding was associated with a higher percentage of “somewhat agree” or lower or “agree”, and a lower percentage of “strongly agree” although none of the differences achieved statistical significance. For example, adjusted results of patient ratings by hospital outpatient volume (S3 Fig) showed a strong gradient between hospital volume quartiles (2^nd^ to 4^th^ quartile since none of the Tier 3 hospitals was of the bottom quartile) and patient ratings in the sense that higher volumes were associated with poorer ratings; however, the 95% confidence intervals of predicted ratings by quartile overlapped.

## Discussion

Using data from a patient experience survey administered at 40 public hospitals in 2014 in Shanghai, China, we found that patients seeking outpatient care at these hospitals provided high ratings for their experience during clinical encounters with a physician. Despite overall limited variation in patient ratings, we found that two items pertaining to physician-patient communication and shared decision-making received less favorable ratings at both general and specialty hospitals. Our findings also suggest that overcrowding of hospital outpatient departments measured by outpatient volume was associated with poorer patient experience during encounters with their physicians and such a relationship was more pronounced at specialty than at general hospitals.

The overall very high ratings of patient experience we found were consistent with findings from a recent outpatient experience survey that included 136 Tier 3 hospitals in all 31 provinces in mainland China [21]. Although patient ratings in that study were based on a 5-point scale and thus not comparable with our results, a similar percentage of patients (50–60%) “strongly agreed” with the statements describing their encounters with their clinicians.

We found poorer patient experience with two items, “Physicians actively asked questions to better understand the patient’s situation” and “Physician provided detailed information regarding the treatment plan including alternative treatments”. We consider them to reflect on both communication and shared decision-making because both require two-way communication and indicate that the physician is taking the initiative to engage the patient in clinical decision-making regarding diagnosis and treatment. Adequate communication is of vital importance to patient self-care and adherence to medication and other treatment regimens [22, 23], and is positively associated with clinical effectiveness [24, 25] and safety [26, 27]. Engagement of patients in the clinical decision-making process by incorporating patient beliefs, concerns, and preferences increases patients’ sense of ownership of their care and was associated with improved treatment adherence and self-management [22, 28, 29]. Poor communication and shared decision-making during brief physician encounters may be especially detrimental for Chinese patients seeking care at Tiers 2 or 3 public hospitals since patients typically do not have a primary care provider and instead receive care from a different physician at each visit (i.e., whoever they happen to see on the day of visit). The poor continuity of care in general, coupled with very limited opportunities of seeking clarification or asking questions outside clinical encounters, greatly adds to the concern. It is thus not surprising that poor communication, rather than malpractice per se, has been identified as the leading cause of medical disputes in China in several recent studies [30, 31].

The compromise in communication and shared-decision making is of heightened concern to patients with complex and multiple problems. Studies in the U.S. have found that visit lengths did not vary substantially with the number or nature of problems a patient had [14, 32]. Rather, with incentives to maintain or increase volumes of care and the need to manage the daily workflow, physicians seemed to adapt to the rule-of-thumb of spending the same amount of time with all patients. As a result, patients with multiple or complex problems may be disproportionately adversely affected. Physician salaries at public hospitals in China fall seriously short of the true value of their time, giving rise to strong incentives to make up the gap by increasing volumes of care and thus increasing revenues to hospitals, which can be partly redistributed to the physicians in the form of bonuses [33]. With the misalignment of financial incentives and the heavy workload, physicians at public hospitals in China may have adapted to the same heuristics or rule-of-thumb of keeping each visit to a similar length.

Our findings are consistent with the hypothesis that overcrowding of hospital outpatient departments (measured by outpatient volume) was associated with poorer patient experience with physician services, with a stronger relationship seen at specialty hospitals, where patient ratings were statistically significantly poorer at hospitals in the top two quartiles in terms of outpatient volume compared to at hospitals in the bottom quartile. It is possible that outpatient volume might be associated with unobserved or unmeasured characteristics of the hospital (e.g., hospital reputation and severity and complexity of medical conditions seen at the hospital), which, in turn, may bias the association between volume and patient experience. For example, hospitals with good reputation have higher volumes, but good reputation by itself is associated with better patient experience, leading to attenuated association between high volume and poor patient experience. The lack of statistical significance in our results for general hospitals and the absence of a clear gradient between specialty hospitals in the 3^rd^ and 4^th^ volume quartiles might be partially accounted for by such biases.

At least two mechanisms may underlie the association between outpatient volume at hospital and patient experience with physician services. First, physicians faced greater pressure to contain the length of visits when there were larger volumes of outpatients that need to be seen within a given shift, leading to possibly shortened contact time. Second, overcrowding of outpatient departments led to longer waiting time during all phases of their visits to the hospital including the initial registration, physician visit, payment, lab tests and imaging (if needed), and pharmacy. The prolonged waiting time (typically 2–3 hours on average at Tier 3 hospitals) contributed to overall poor patient experience and could have adversely affected their ratings of experience with physician services.

The lack of association between morning (vs. afternoon) visits and patient ratings at general hospitals could reflect the fact that an overwhelming majority of visits at Tier 1 hospitals were in the morning and visits to Tier 1 hospitals were of higher ratings on average. Day of the week seemed to have made a difference (in the direction consistent with our hypothesis) at general hospitals (albeit without statistical significance), but not at specialty hospitals probably because outpatient volumes at specialty hospitals in Shanghai (all Tier 3) did not differ substantially by day of week.

Our findings highlight the negative impacts on patient experience associated with the concentration of outpatient visits at Tiers 2 and 3 hospitals, a prominent issue that China’s public hospital reform intends to address [34]. In our sample of general hospitals, the interquartile range of daily outpatient visits in the first half of 2014 was 1,431 (pertaining to a Tier 1 hospital) to 8,834 (pertaining to a Tier 3 hospital). Despite strategies to build up the capacities of primary and preventive care at community health centers (Tier 1 hospitals) [34], the public’s trust in the quality of care delivered at community health centers remains low regardless of the nature or complexity of the conditions patients are seeking care for. Our data suggest that two-thirds of all outpatients to Tier 3 general hospitals and almost three-quarters of all outpatients to Tier 2 hospitals in our study were returning patients seeking care for existing conditions. It is likely that some of these visits could have been effectively managed at community health centers.

Two promising strategies have emerged in recent efforts to divert substantial volumes of ambulatory care from Tiers 2 and 3 hospitals to community health centers. One is a primary care contract system where older patients and patients with chronic conditions are encouraged to “contract with” a community health center to meet their needs for primary care in return for priority referral to partnering Tier 2 or 3 hospitals, filling and refilling their chronic condition medications at the center (even if prescribed at higher-tier hospitals), and/or lower out-of-pocket payment for care at Tier 2 or 3 hospitals if referred by the community health center [35]. This strategy addresses demand-side factors (patient preferences for hospitals of different tiers) and aims at establishing community health centers as the primary care gatekeeper. The other strategy is a supply-side effort aimed at vertical integration of medical care resources by forming strategic alliances among hospitals of different tiers. In addition to facilitating two-directional referrals of patients among participating hospitals to achieve efficient use of resources, it is hoped that hospitals of higher tiers could support hospitals of lower tiers by direct provision of care in low-tier settings, tele-health, and training and mentoring of clinicians in community health centers [36]. However, the effectiveness of these strategies might be limited in absence of major changes to current incentive systems for hospitals and physicians, one that forces hospitals to become profit centers and rewards volume of care [33].

Our study has a few important limitations. All hospitals included in our study were located in Shanghai, one of the economically most developed areas in China that also has abundant resources for medical care compared to the rest of China. In particular, it is an exception to have a large number of Tiers 3 and 2 hospitals in a small geographic area such as the central city of Shanghai. Thus, our findings may not be generalizable to hospitals in other areas, although, as described, the distribution of patient ratings for clinical encounters in our study was quite consistent with what was reported in a recent national study [21]. On the other hand, outpatient department overcrowding at Tiers 2 and 3 hospitals is norm rather than exception across China, and, through similar mechanisms discussed, could have compromised patient experience with physician services. In our study, patient responses to survey questions heavily concentrated in the top two categories, leaving the bottom four categories combined accounting for about 10% of all responses. This could reflect systematic biases in responses, for example, it may be culturally and socially desirable to provide a favorable rating. Because of this lack of variation, we chose to report and discuss all findings rather than focusing on results that achieved statistical significance.

## Conclusions

Our analysis of data from a recent public hospital outpatient experience survey in Shanghai, China indicated overall favorable patient experience with physician service, but also identified physician-patient communication and shared decision-making as areas with deficits. In addition, we found hospital outpatient department overcrowding to be a possible contributor to poor patient experience with physician services. Fundamental changes in incentive systems and innovative policies and strategies are needed to effectively divert outpatients with chronic and less complex conditions to community health centers, which, in turn, will likely improve patient experience with outpatient care at public hospitals.

## Supporting information

S1 Text Validity and reliability tests of the patient experience survey. (DOCX)

S1 Fig Patient ratings by morning vs. afternoon visits: Tier 3 general hospitals. (TIF)

S2 Fig Patient ratings by Monday vs. Tuesday-Friday visits: Tier 3 general hospitals. (TIF)

S3 Fig Patient ratings by quartile of ambulatory volume of hospital: Tier 3 general hospitals. (TIF)
