# Supplementary material for: Patient experience with outpatient encounters at public hospitals in Shanghai: Examining different aspects of physician services and implications of overcrowding
Source: PLoS One. 2017 Feb 16;12(2):e0171684. doi: 10.1371/journal.pone.0171684 (PMC5312958; doi:10.1371/journal.pone.0171684)
Supplement: S1 Text — (DOCX) [file pone.0171684.s001.docx]

# S1 Text. Validity and reliability tests of the patient experience survey.

Using data from the 2013 pilot test of the patient experience survey, we conducted item analysis (Cronbach’s alpha), exploratory factor analysis, and confirmatory factor analysis (structural equation model) to test the reliability and validity of the questionnaire. In confirmatory factor analysis, we used modification indices (MIs) to modify the model and used the fitness indices to select the best model from alternative models. Based on these analyses, four dimensions based on 37 items were ultimately included in the patient experience survey.

Table 1 below presents the goodness-of-fit test statistics for the 2013 data (pilot) and the 2014 data (used in this study); Table 2 presents the internal validity measures for the entire questionnaire and for the 4 individual dimensions.

Table 1. Tests of Goodness of Fit

| Parameters | 2013 | 2014 |
| --- | --- | --- |
| Fit Function | 2.0834 | 2.5286 |
| Chi-Square | 11600.4911 | 4943.4992 |
| Chi-Square DF | 771 | 661 |
| χ^2^/DF | 15.0460 | 7.4788 |
| Pr > Chi-Square | <.0001 | <.0001 |
| Standardized Root Mean Square Residual (SRMR) | 0.0412 | 0.0346 |
| RMSEA Estimate | 0.0502 | 0.0576 |
| RMSEA 90% Lower Confidence Limit | 0.0494 | 0.0561 |
| RMSEA 90% Upper Confidence Limit | 0.0510 | 0.0591 |
| Bentler's Comparative Fit Index (CFI) | 0.9329 | 0.9378 |
| James, Mulaik, & Brett (1982) Parsimonious NFI (PNFI) | 0.8730 | 0.8734 |

Table 2. Internal validity measures

| Overall and Dimension | Cronbach's α | |
| --- | --- | --- |
|  | 2013 | 2014 |
| overall | 0.9679 | 0.9791 |
| facilities and equipment | 0.8506 | 0.8947 |
| physician services | 0.9627 | 0.9694 |
| non-physician services | 0.8959 | 0.9492 |
| ancillary processes and effectiveness | 0.8994 | 0.9260 |
