# Supplementary figures and images for: Patient experience with outpatient encounters at public hospitals in Shanghai: Examining different aspects of physician services and implications of overcrowding
Source: PLoS One. 2017 Feb 16;12(2):e0171684. doi: 10.1371/journal.pone.0171684 (PMC5312958; doi:10.1371/journal.pone.0171684)

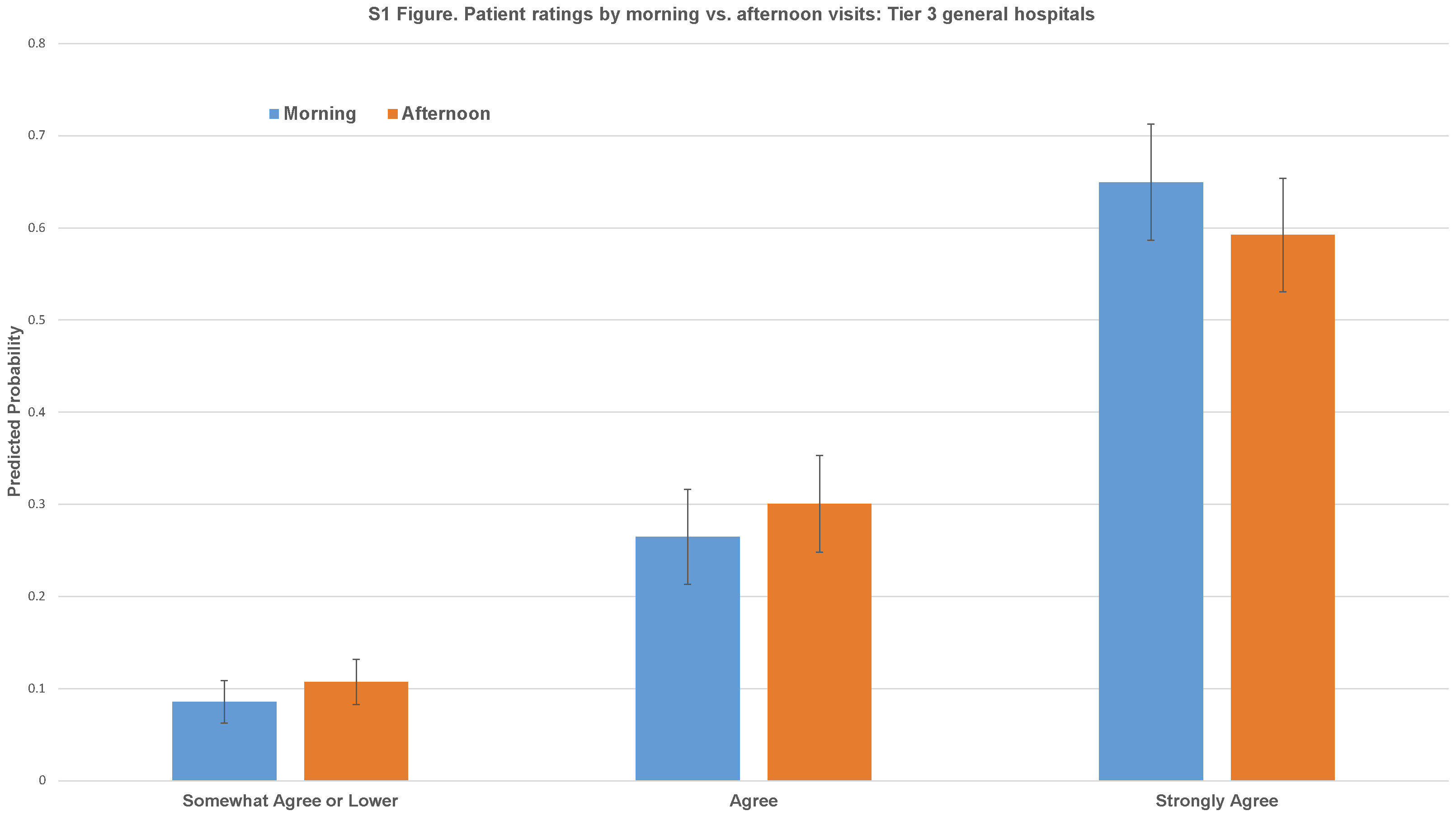

Supplement: S1 Fig — (TIF) [file pone.0171684.s002.tif]

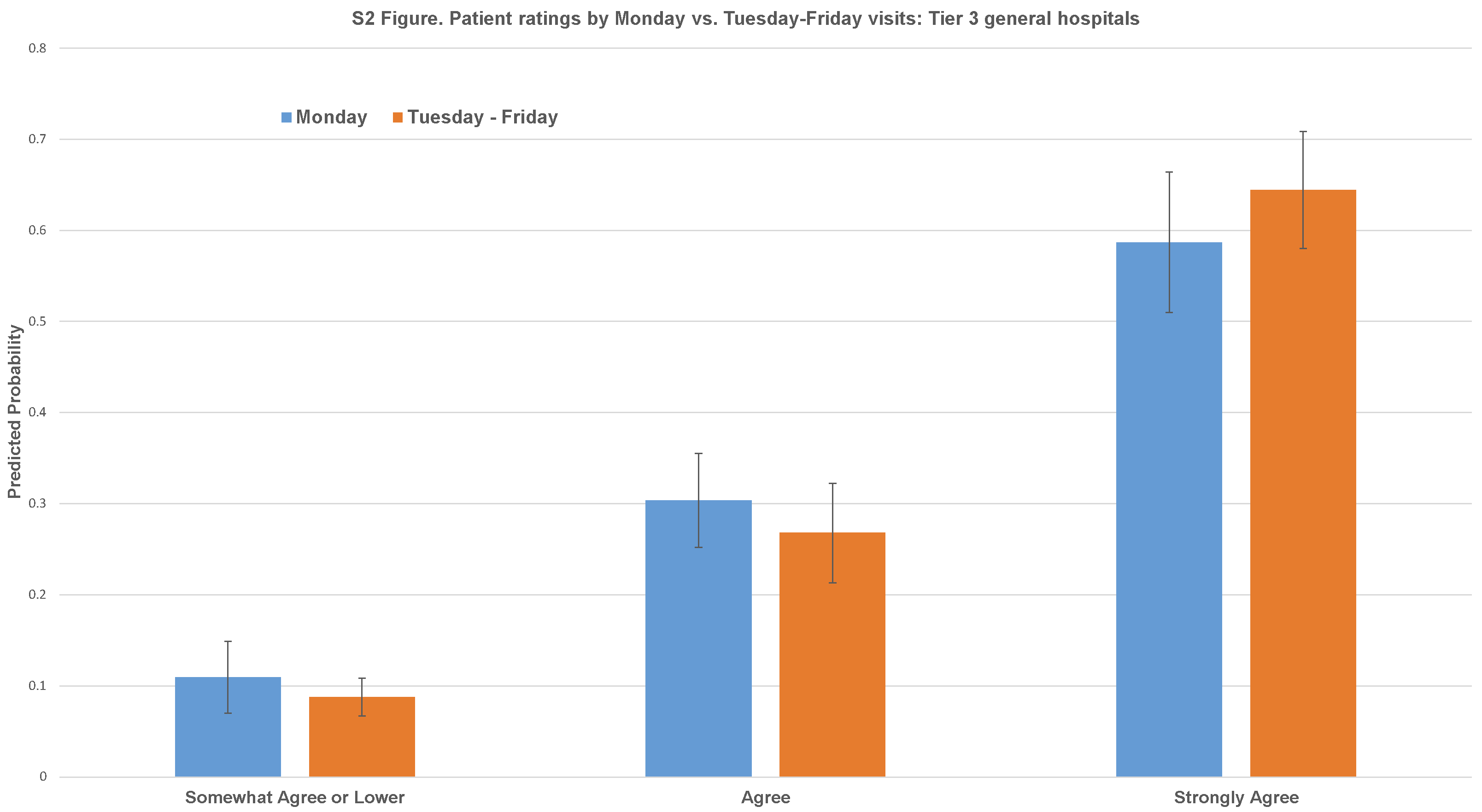

Supplement: S2 Fig — (TIF) [file pone.0171684.s003.tif]

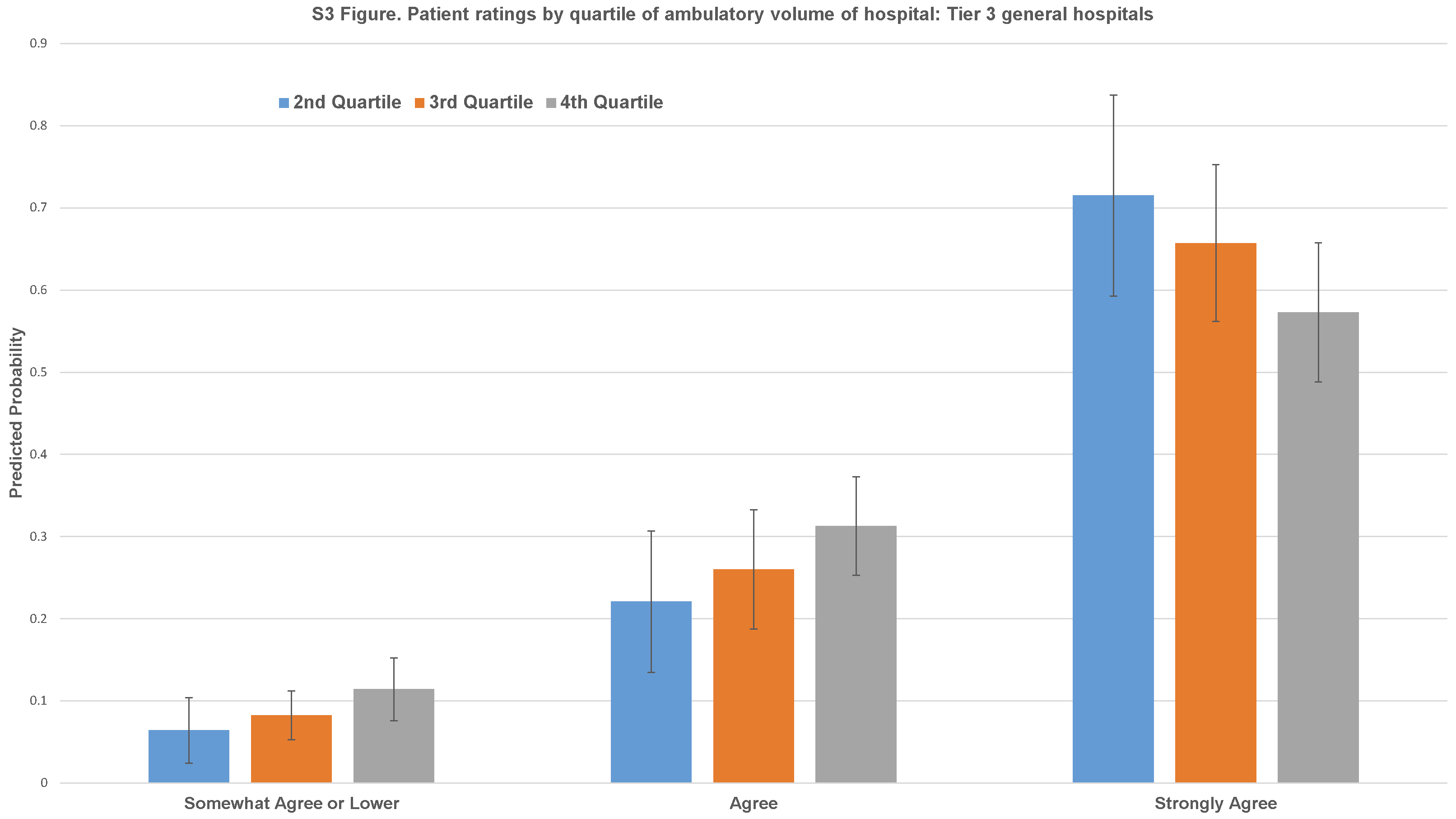

Supplement: S3 Fig — (TIF) [file pone.0171684.s004.tif]
